# Automated Detection and Localization of Synaptic Vesicles in Electron Microscopy Images

**DOI:** 10.1523/ENEURO.0400-20.2021

**Published:** 2022-01-19

**Authors:** Barbara Imbrosci, Dietmar Schmitz, Marta Orlando

**Affiliations:** 1 German Center for Neurodegenerative Diseases (DZNE) Berlin, Berlin 10117, Germany; 2 Charité – Universitätsmedizin Berlin, corporate member of Freie Universität Berlin and Humboldt-Universität zu Berlin, and Berlin Institute of Health, Berlin 10117, Germany; 3 NeuroCure Cluster of Excellence, Berlin 10117, Germany; 4 Bernstein Center for Computational Neuroscience (BCCN) Berlin, Berlin 10115, Germany; 5 Einstein Center for Neurosciences (ECN) Berlin, Berlin 10117, Germany; 6 Max-Delbrück-Centrum (MDC) for Molecular Medicine, Berlin 13125, Germany

**Keywords:** automated detection, convolutional neural networks, image analysis, machine learning, synaptic vesicle

## Abstract

Information transfer and integration in the brain occurs at chemical synapses and is mediated by the fusion of synaptic vesicles filled with neurotransmitter. Synaptic vesicle dynamic spatial organization regulates synaptic transmission as well as synaptic plasticity. Because of their small size, synaptic vesicles require electron microscopy (EM) for their imaging, and their analysis is conducted manually. The manual annotation and segmentation of the hundreds to thousands of synaptic vesicles, is highly time consuming and limits the throughput of data collection. To overcome this limitation, we built an algorithm, mainly relying on convolutional neural networks (CNNs), capable of automatically detecting and localizing synaptic vesicles in electron micrographs. The algorithm was trained on murine synapses but we show that it works well on synapses from different species, ranging from zebrafish to human, and from different preparations. As output, we provide the vesicle count and coordinates, the nearest neighbor distance (nnd) and the estimate of the vesicles area. We also provide a graphical user interface (GUI) to guide users through image analysis, result visualization, and manual proof-reading. The application of our algorithm is especially recommended for images produced by transmission EM. Since this type of imaging is used routinely to investigate presynaptic terminals, our solution will likely be of interest for numerous research groups.

## Significance Statement

The analysis of synaptic vesicles provides important insights toward the understanding of synaptic transmission and plasticity mechanisms. However, up to date, this analysis is still a very time-consuming manual process. In the present study we present a user-friendly algorithm, mainly based on convolutional neural networks (CNNs), for automating the detection of synaptic vesicles in electron micrographs. This approach allows faster and more standardized analyses.

## Introduction

In the presynaptic terminal, synaptic vesicle abundance (Patzke et al., 2019), clustering (Milovanovic et al., 2018; Pechstein et al., 2020), recycling (Kononenko and Haucke, 2015; Tagliatti et al., 2016; Ackermann et al., 2019), and turn-over (Vijayan and Verstreken, 2017) are pivotal indicators of synaptic function and are altered in aging (Maglione et al., 2019) and in neurologic diseases such as Parkinson’s disease (Diao et al., 2013) or Alzheimer’s disease (Marsh and Alifragis, 2018). Synaptic vesicles are held in the proximity of release sites by scaffolds and molecular bridges and the distance between synaptic vesicles and the active zone is an important parameter that regulates neurotransmitter release (Imig et al., 2014; Chang et al., 2018; Quade et al., 2019). The distribution of vesicles is controlled by activity (Chi et al., 2001; Pechstein and Shupliakov, 2010) and is thought to sustain short (Vandael et al., 2020) and long-term plasticity (Rey et al., 2020; Orlando et al., 2021).

To visualize synaptic vesicle release and trafficking, fluorescence microscopy techniques are available (Kavalali and Jorgensen, 2014). Nevertheless, since synaptic vesicles are very small organelles, having a diameter of 30–40 nm, electron microscopy (EM) is the state-of-the-art method for the analysis of their number, area, and distribution in synapses.

The study of synaptic vesicles localization is of major scientific interest in the field of neurobiology. However, the manual identification of vesicles is a tedious task, that becomes particularly time-consuming for scientists that investigate giant synapses such as calyx of held synapses (Qiu et al., 2015), cerebellar mossy fibers (Falck et al., 2020) or hippocampal mossy fibers boutons (hMFBs; Rollenhagen and Lübke, 2010) where thousands of vesicles can be found. Moreover, morphologic manual analysis can differ depending on the researcher performing it, because of individual subjective biases.

Automated methods for the detection of synaptic vesicles are therefore needed to increase the analytical throughput, to reduce manual labor and to improve standardization.

In the last years, we experienced a rapid advancement in the automated analysis of natural images thanks to success of deep convolutional neural networks (CNNs; Krizhevsky et al., 2012). In fact, CNNs architectures have already been proposed in the late 1980s, but only recently, with the availability of large amount of labeled data and the development in computing power, they gained momentum and have started to be used in a great variety of applications, including object detection tasks (Rawat and Wang, 2017).

In the field of neuroanatomy, CNNs have been proven to be an effective method for automating the segmentation of neuronal structures (Arganda-Carreras et al., 2015).

Several studies in the field of connectomics have successfully employed CNNs to compute large-scale 3D reconstructions of neuronal circuits (Cireşan et al., 2012; Ronneberger et al., 2015; Januszewski et al., 2018). A first successful attempt to identify synaptic vesicles in presynaptic terminals required tomographic 3D reconstructions (Kaltdorf et al., 2017). EM tomograms, while providing detailed 3D information on single vesicles, are nevertheless lengthy to acquire.

In the present study, we exploited the power of CNNs and built a model capable of recognizing synaptic vesicles in electron micrographs. Our CNN model, combined with a connected-component labeling and clustering-based segmentation algorithm, efficiently detects and localizes vesicles from images of presynaptic terminals. Our algorithm performed well on transmission EM images of synapses of different species, with different resolution (tested pixel size ranging from ∼0.7 to ∼5 nm) and prepared with different techniques. The results were optimal when vesicles were sharp and their lumen and membrane were visible.

Since the algorithm worked well across these different images, and since the shape and dimension of synaptic vesicles varies only minimally, across species, brain areas, and different fixation protocols, we are confident that our model can be directly applied without the need to be retrained.

Furthermore, to offer a simple and flexible tool to researchers, we developed a graphical user interface (GUI) that offers a step-by-step guidance for analyzing, displaying and proof-reading the results. This GUI allows the analysis of multiple images at once (as long as they have the same resolution) and provides the results automatically in an excel file. Furthermore, it offers the possibility to easily visualize and correct the results (both by adding missed vesicles or deleting erroneously predicted vesicles).

We are confident that our tool can significantly increase the efficiency of synaptic vesicle analysis and reduce the workload of research groups focusing on the study of presynaptic structure and function.

## Materials and Methods

### Preparation of acute brain slices for EM imaging

All animal experiments were approved by the animal welfare committee of the Charité Universitätsmedizin Berlin and the Landesamt für Gesundheit und Soziales Berlin, Germany (permit #T0100/03). Three postnatal day (P)27–P29 male WT C57BL/6N mice were anesthetized with isoflurane, decapitated for a project on structural plasticity (Orlando et al., 2021). Brains were quickly removed and placed in ice-cold sucrose-artificial CSF (s-ACSF) containing the following: 50 mm NaCl, 25 mm NaHCO_3_, 10 mm glucose, 150 mm sucrose, 2.5 mm KCl, 1 mm NaH_2_PO_4_, 0.5 mm CaCl_2_, and 7 mm MgCl_2_. All solutions were saturated with 95% O_2_/5% CO_2_ (v/v), pH 7.4. Sagittal slices 350 μm thick were cut with a VT1200S vibratome (Leica) in ice cold s-ACSF solution and stored submerged in s-ACSF for 30 min at 35°C and subsequently stored at room temperature in ACSF containing the following: 119 mm NaCl, 26 mm NaHCO_3_, 10 mm glucose, 2.5 mm KCl, 1 mm NaH_2_PO_4_, 2.5 mm CaCl_2_, and 1.3 mm MgCl_2_ saturated with 95% O_2_/5% CO_2_ (v/v), pH 7.4. No more than 6 h after the preparation, acute slices were immersed in a solution containing 1.2% glutaraldehyde in 66 mm Na cacodylate buffer for 1 h at room temperature. After washes in 0.1 m Na cacodylate buffer slices were then postfixed in 2% OsO_4_ in dH_2_O for 1 h at room temperature. Slices were then washed and en bloc stained with 1% uranyl acetate in dH_2_O and dehydrated in solutions with increasing ethanol concentration. Final dehydration was obtained incubating slices in Propylene oxide and then the infiltration of Epoxy resin was obtained by serial incubations in increasing resin/propylene oxide dilutions. Samples have been finally flat embedded in Epon (#E14120-DMP, Science Services) for 48 h at 60°C. The stratum lucidum in the CA3 region of the hippocampus was identified using a light microscope and 70-nm sections of these regions of interest were cut with an Ultracut UCT ultramicrotome (Leica) equipped with an Ultra 45 diamond knife (Diatom) and collected on pioloform-coated copper slot grids (#EMS2010-Cu, Science Services). Synapses were identified and imaged using an EM 900 Zeiss Transmission Electron Microscope, or a Tecnai G2 20 (FEI Thermo Fisher Scientific; RRID: SCR_021365) operated at 80–120 keV and equipped with a Proscan 2K Slow-Scan CCD-Camera (Carl Zeiss) and a Veleta 2K x 2K CCD camera (Olympus), respectively.

### Preparation of hippocampal cultures for EM imaging

Primary neuronal hippocampal cultures were prepared as previously described (Orlando et al., 2019). Briefly, primary neuronal cultures were generated from both sexes of postnatal mice from the C57/BL6N strain aged P0–P2 (permit #T0220/09). Brains were removed and placed in 4°C cooled HBSS (Invitrogen). Hippocampi were carefully dissected out and placed in Neurobasal-A medium supplemented with B27, Glutamax (all from Invitrogen), and penicillin/streptavidin (Roche; full-NBA) at 37°C in a heated shaker. Full-NBA was replaced with DMEM (Invitrogen), supplemented with 1 mm CaCl_2_ and 0.5 mm EDTA (enzyme solution), containing papain (22.5 U/ml; CellSytems GmbH) and incubated for 45–60 min. The digestion was stopped by removing the enzyme solution and replacing it with an inactivating solution of DMEM supplemented with albumin (2.5 mg/ml) and trypsin inhibitor (2.5 mg/ml; both Sigma-Aldrich). The inactivating solution was removed after 5 min and replaced with full-NBA. Tissue was dissociated mechanically, and cells were counted on a Neubauer chamber. Dissociated cells were plated on 6 mm carbon-coated sapphire disks (Wohlwend) at a density of ∼250 cells/mm^2^. At 13–15 d of growth *in vitro* (DIV), primary hippocampal neurons grown on sapphire discs were transferred to the chamber of a high-pressure freezing machine (EM ICE (RRID: SCR_021367) or HPM 100 (RRID: SCR_021366), Leica Microsystems) and cryo-fixed in extracellular solution containing the following: 140 mm NaCl, 2.4 mm KCl, 10 mm HEPES (Merck), 10 mm glucose (Carl Roth), 2 mm CaCl_2_ (Sigma-Aldrich), and 4 mm MgCl_2_ (Carl Roth); 300 mOsm; pH 7.4. Cryo-fixation was followed by freeze-substitution in anhydrous acetone containing 1% glutaraldehyde, 1% osmium tetroxide and 1% milliQ water in an automated freeze-substitution device (AFS2, Leica). The temperature was kept for 5 h at −90°C, brought to −20°C (5°C/h), kept for 12 h at −20°C and then brought to +20°C (5°C/h). Once at room temperature, samples were en bloc stained in 0.1% uranyl acetate in acetone, infiltrated in increasing concentration of Epoxy resin (Epon 812, EMS) in acetone and embedded in pure resin for 48 h at 65°C. Sapphire discs were removed from the cured resin block by thermal shock; 50-nm-thick sections were obtained using an Ultracut UCT ultramicrotome (Leica) equipped with an Ultra 45 diamond knife (Diatome) and collected on formvar-coated 200-mesh formvar-coated copper grids (#EMS200-Cu, Science Services). Sections were counterstained with uranyl acetate and lead citrate and synapses were identified and imaged using a Tecnai G2 20 (FEI Thermo Fisher Scientific) operated at 80–120 keV and equipped with a Veleta 2K x 2K CCD camera (Olympus). Images of chemically-fixed cultured hippocampal neurons where obtained with a JEM-1011 (JEOL) transmission EM. For details on the sample preparation see https://www.protocols.io/view/chemical-fixation-and-embedding-of-cultured-cells-bwsbpean.

### Development of a vesicle classifier

All programming was done with python 3.6 or python 3.7 (Python Software Foundation; https://www.python.org/) either using a business-oriented laptop with a Windows 7 Professional operating system or a High Performance Compute (HPC)/GPU Server (GPU: NVIDIA GeForce RTX 2080) with a Ubuntu 18.04 LTS or an openSUSE Leap 15.2 operating system.

To train the image classifier we used 21 electron micrographs, of which 19 images of MFBs from acute hippocampal slices of three mice and two images of small synapses from cryo-fixed hippocampal neurons from one litter/culture (dataset train 1, Table 1). From these images, we generated 34,805 patches (40 × 40 pixels, 90.8 × 90.8 nm), and we manually labeled them as either containing or not containing a vesicle. This training dataset had a ratio of 2.84 between classes non containing (negative) or containing (positive) a vesicle. We used this slightly unbalanced dataset because a perfectly balanced one yielded slightly worse results (results not shown) and adding negative examples improved it. Among negatives examples, we also included black patches (4184). This allows users to use a black mask in case they want to exclude a part of an image from the analysis. We further applied data augmentation using the torchvision python library (https://pytorch.org/), to increase the variability of the training dataset, since this technique has been proven to increase model performance and reduce overfitting (Shorten and Khoshgoftaar, 2019). We employed spatial (10% rotation), color augmentation (20% variation in brightness, contrast and saturation) and Gaussian noise (mean 0 and σ 0.1 with a probability of 0.2 and mean 0, σ 0.05 with a probability of 0.1). We evaluated the model by averaging the results over four rounds of cross-validation performed by further splitting the training dataset into training (75%) and validation (25%) subsets.

**Table 1:** Description of the datasets

Dataset	Total images	Acute slices	Neur. cultures (cryo/chem.fix.)	Patches/full images	Usage
Train 1	21	19	2/0	Patch. 34805	Train 1° cl.
Test 1	6	4	2/0	Patch. 4209	Test 1° cl.
Train 2	16	10	6/0	Patch. 6245	Train 2° cl.
Test 2	8	5	3/0	Patch. 1912	Test 2° cl.
Test final	27	11	7/9	Full images 27	Evaluation
External	10	0	2/8	Full images 27	Evaluation

Description of the datasets used to train and test the first and second (refinement) classifiers and for evaluating the final performance of the model. The term “acute slices” refers to images of hMFBs from chemically-fixed acute hippocampal slices, “neur. cultures” refers to images of small hippocampal synapses from either cryo-fixed or chemically-fixed cultured neurons.

To test the performance of the classifier on patches, we used patches (4209) obtained from six different images, of which four images of MFBs from acute hippocampal slices of two mice and two images of small synapses from cryo-fixed hippocampal neurons from one litter/culture (dataset test 1; Table 1). Similar to the training dataset, the testing dataset was also slightly unbalanced, having a ratio of 2.94 between negative and positive classes, and did also contain black patches within the negative examples (146).

The classifier was built on pytorch, an open-source machine learning library for python (https://pytorch.org/; Paszke et al., 2019). The network consists of four convolutional layers followed by one 2 × 2 max pooling layer and three fully connected layers. All convolutional layers have convolutional filters of size 7 × 7 and all inputs to the convolutional layers are padded with two zeros pixels on both sides. We applied the Rectified Linear Unit (ReLU) activation function in all layers and added dropout between the fully connected layers as well as between the last two convolutional layers to regularize the network (Srivastava et al., 2014). To train our classifier, we used the cross-entropy loss function, the ADAM optimization algorithm (Kingma and Ba, 2017), and set the learning rate at 0.0002.

To detect and localize the large number of vesicles present in an image from a presynaptic terminal, we fed the CNN with 40 × 40 image patches cropped from the original EM images with a sliding window with a 4 × 4 pixels stride. Image padding was applied to optimize the detection of vesicles at the edge of an image. This consists of adding 20 pixels with zeros at each side of the images before letting them being analyzed by the classifier.

Furthermore, to guarantee a good vesicle prediction on images with different resolutions, we included a step to rescale input images to have the same pixel size as the one used for training the network (2.27 nm).

For every iteration, our classifier assigned to the corresponding pixel the probability to belong to a vesicle. A patch corresponding to the coordinates −20:+19,−20:+19 with respect to the evaluated pixel was used by the classifier to extract information. As output, we obtained a probability map expressing the likelihood of each pixel in the micrograph to belong to a vesicle. On this output probability (pr.), we applied a cutoff value of 0.5, such that if a pixel was predicted to be within a vesicle (pr. ≥ 0.5) we set pr. = pr.; otherwise, we set pr. = 0. The probability map was then resized to match the size of the original image, using a bilinear interpolation and smoothened, using a low-pass filter, by convolving the image with a normalized box filter with a 3 × 3 kernel size. Finally, the range of pixel values was converted from 0 to 1 to 0 to 255. A probability map tells how likely it is that each pixel in an image belong to an object rather than to the background but does not distinguish single objects. In order to identify separated objects (potential synaptic vesicles) we applied a threshold-based segmentation and a connected-component labeling algorithm on the probability map with a 3 × 3 structuring element with a squared connectivity equal to one. Occasionally the objects distinguished by the connected-component labeling algorithm contained a small group of vesicles rather than a single one. Therefore, to identify and separate each vesicle, we applied a k-means clustering algorithm on the detected “objects”.

To make the most accurate guess on the number of vesicles present in each “object,” we checked the number of peaks in the portions of the probability map corresponding to each “object.” The number of peaks with a Euclidian distance larger than 34 nm (which represents roughly the diameter of a vesicle) turned out to be a very good estimator of the number of vesicles and it was therefore used to define the number of clusters in the k-means clustering algorithm. Finally, we set a threshold of 330 nm^2^ (corresponding to the area of 64 pixels) and excluded clusters with an area smaller than this value, since very small clusters likely correspond to false positives. This threshold was set to slightly higher values in images with a relatively low resolution, since the large rescaling of the probability map is likely to generate larger clusters (pixel size ≥ 2.3 nm: threshold 407 nm^2^; pixel size ≥ 3.3 nm: threshold 484 nm^2^; pixel size ≥ 4.3 nm: threshold 562 nm^2^; pixel size ≥ 5.3 nm: threshold 639 nm^2^; pixel size ≥ 6.3 nm: threshold 716 nm^2^). The validity of this approach, namely, applying a k-means clustering algorithm and setting a threshold for cluster dimension, in improving the performance of our model is shown in Figure 2*D–F* and described in Results.

The sequential application of the described CNN, connected-component labeling and clustering-based segmentation algorithm was very effective in detecting presynaptic vesicles. Indeed, we observed a very low number of false negatives. However, false positives were numerous (for details, see Results and Fig. 2*B*). To reduce these, we included a final step: we let patches, with a size of 80 × 80 pixels, centered around the detected vesicles to be evaluated a second time by an additional CNN. This was performed after padding the images by adding 40 pixels with zeros at each side. This second CNN, that we named refinement classifier, has the same network architecture, loss function and optimization algorithm as the first one. It only differs in the learning rate: 0.0004 instead of 0.0002. The detected vesicles produced as final output by our algorithm are all the ones predicted as positives by this refinement classifier.

To train this second refinement classifier we used 16 electron micrographs, of which 10 images of MFBs from acute hippocampal slices from three mice and six images of small synapses from cryo-fixed hippocampal neurons from one litter/culture (dataset train 2; Table 1). All images, except one, were different from the ones used to train the first classifier. From these images we generated 6245 patches (80 × 80 pixels, 181.6 × 181.6 nm) and we manually labeled them as either containing or not containing a vesicle. As the first training dataset, this was also slightly unbalanced, having a ratio of 2.18 between negative and positive classes. Similarly, to our first model, we applied data augmentation as a strategy to increase the variability of the training dataset. We employed spatial (10% rotation), color augmentation (20% variation in brightness, contrast and saturation), and Gaussian noise (mean 0, σ 0.1 with a probability of 0.1).

Finally, to test the refinement classifier we used 1912 patches obtained from eight different images, of which five images of MFBs from acute hippocampal slices from two mice and three images of small synapses from cryo-fixed hippocampal neurons from one litter/culture (dataset test 2; Table 1). This second testing dataset had a ratio of 2.32 between negative and positive classes.

Our algorithm produces, as output, an excel file containing a summary result sheet with the total number of detected vesicles for each analyzed image and then a separate sheet for each image containing the vesicle position, the distance to the nearest vesicle (nearest neighbor distance; nnd) in nm and the estimated area for each detected vesicle in nm^2^. The vesicle position was measured as the *x*, *y* coordinates of the center of the cluster obtained after applying the connected-component labeling and clustering-based segmentation algorithm on the probability map produced by the CNN. The nnd was calculated as the shortest Euclidian distance from the position of one vesicle to the position of all remaining ones. To calculate the vesicles area, we took advantage of two facts: (1) that pixels corresponding to the membrane delimiting the vesicles have generally lower values (darker) with respect to the pixels corresponding to the vesicles lumen and to the vesicles immediate surroundings (brighter) and (2) that vesicles shape (elliptical-circular) and dimension (diameter circa between 30 and 55 nm) is relatively stereotypical across species, brain areas and different fixation and imaging protocols. Briefly, we created a 40 × 40, 0–1 matrix, and we drew elliptical or circular shapes on it with the thickness of three pixels (6.81 nm) and with different radius and ratios (major/minor axis) assigning the value of one to the pixels corresponding to the drawn shape and zero otherwise. Then, we multiplied this matrix with a 40 × 40 patch centered at each detected vesicle (so that the position of the detected vesicle corresponded to the pixel in the 21st column and 21st row of the image patch), and calculated the average pixel value on the elliptical shape of the so obtained matrix. We repeated this measurement trying all combinations of elliptical-circular shapes with radius-axis comprised between 7 and 12 pixels (15.89–27.24 nm) with the only condition that the major and minor axis could not differ by more than four pixels (9.08 nm). We also repeated the measurement moving the matrix up to three pixels in all directions (up, down, left, right) since the initially determined position may not always correspond to the exact center of a vesicle. Since occasionally the pixels corresponding to the vesicle’s membrane were not homogeneously dark, we also added a term to penalized asymmetry, namely, 0.03*SD of the mean pixel values for the four quadrants of the matrix obtained after multiplication. The elliptical shape and position obtaining the lowest intensity value, calculated as described above, was considered the one delimiting the vesicle. We finally calculated the vesicles area, knowing the major and minor radius with the following formula: major radius * minor radius * π * area of one pixel in nm^2^.

### Quantification of the performance of the algorithm

To quantify the performance of our algorithm in detecting presynaptic vesicles, we used electron micrographs from three different preparations: hMFBs from chemically-fixed acute hippocampal slices, small hippocampal synapses from cryo-fixed cultured neurons and small hippocampal synapses from chemically-fixed cultured neurons. This dataset (dataset test final, Table 1) was composed of entirely different images than those used to train (dataset train 1, train 2) and test (dataset test 1, test 2) the first and the refinement classifier. Moreover, we selected various publicly available images to further test the ability of our model to generalize (dataset external Table 1).

The performance was evaluated by calculating precision, recall and F1 score with the following formulae:

$$precision=\frac{true\;positives}{true\;positives+false\;positives}$$

$$recall=\frac{true\;positives}{true\;positives+false\;negatives}$$

$$F1score=\frac{2*precision*recall}{precision+recall}.$$

The vesicles predicted by the algorithm were compared with human annotations performed by two different postdoctoral researchers. In the figures where human annotations are graphically displayed on an EM image, the annotations from one of the two postdoctoral researchers are used. Precision, recall and F1-score were calculated using either researcher’s results as ground truth and then by averaging the two values.

The following procedure was used to determine true positives, false positives and false negatives.

We considered true positives only those cases where we could find a 1–1 association between ground truths (human annotations) and algorithm predictions.

To achieve this, we first selected ground truth/prediction pairs, by running a loop over all ground truths and searching, for each, its nearest prediction. If the Euclidean distance, between ground truth and the closest prediction, fell within 28.89 nm, we considered the two coordinates a pair. The chosen distance corresponds to the lower boundary of the diameter of a synaptic vesicle. This distance was set, because the human annotation and the algorithm prediction, referring to the same vesicle, are often found in the very close proximity (within a distance corresponding to the diameter of a vesicle) but rarely in the exact same location. All ground truths which remained unpaired were considered false negatives. The same procedure was repeated by running a loop over all predictions. We used the averaged count of true positives (resulting from looping over predictions and ground truths). All predictions which remained unpaired were considered false positives.

Finally, we also compared the number of manually detected vesicles (as the average between the vesicle counts from the two human annotations) with the number of vesicles detected by our algorithm (Extended Data Fig. 3-1).

**Figure 1.**
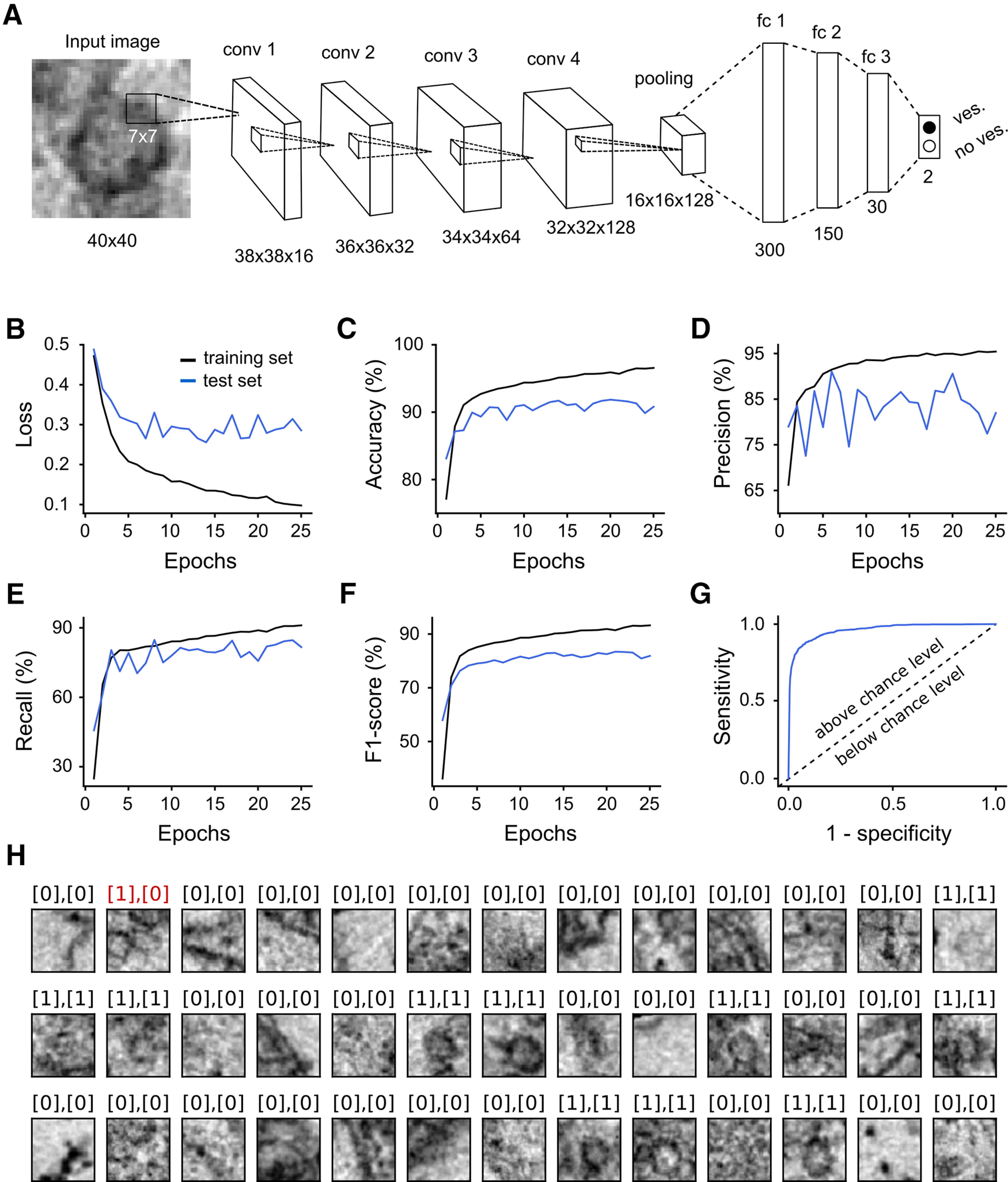
Architecture and performance of the vesicle classifier. ***A***, Architecture of the CNN and diagrams showing the (***B***) cross-entropy loss, (***C***) accuracy, (***D***) precision, (***E***) recall, (***F***) F1-score, and (***G***) ROC curve on the training and test dataset (black and blue, respectively). ***H***, Prediction of the vesicle classifiers on 39 image patches from the test dataset. The number in the first square brackets, on top of each image, represents the label assigned manually whereas the number in the second square brackets represents the prediction done by the classifier. The value 0 indicates that the label/prediction was negative (no vesicle) while the value 1 indicates a positive label/prediction (vesicle). In this representative example, 38 out of 39 images were predicted correctly. Red and black colors are used to indicate wrong and correct predictions, respectively.

10.1523/ENEURO.0400-20.2021.f3-1 Extended Data Figure 3-1 Comparison between synaptic vesicles count detected by humans and by the algorithm. Number of vesicles detected manually and by the algorithm in micrographs from (***A***) hMFBs from chemically fixed acute hippocampal slices, (***B***) small hippocampal synapses from cryo-fixed cultured neurons, and (***C***) small hippocampal synapses from chemically-fixed cultured neurons. Download Figure 3-1, TIF file.

### Vesicles detection using ilastik

We used ilastik, an already available machine learning-based algorithm for analysis of (bio)images (Berg et al., 2019), to validate the results of our model.

To detect synaptic vesicles with ilastik we used two workflows, sequentially: Cell Density Counting and Object Classification (Inputs: Raw Data, Pixel Prediction Map). We chose the Cell Density Counting workflow since it is suitable for circular objects of the same size (as synaptic vesicles). We trained the algorithm by using manual annotations from the same images employed for training our first classifier. Cell Density Counting produces the density of objects as output, therefore, to be able to use the Object Classification workflow we converted the density images into probability. To do so, for each pixel, x, in a density image (image) we calculated the probability as a new pixel value, x_new_, as following:

$$x_{new}=\frac{\text{x}-\text{ min}\left(image\right)}{\max\left(image\right)-\text{min}(image)}.$$

We then used the same images employed for testing our first classifier to tune some parameters (threshold and size filter) to optimize the Object Classification task (vesicles vs other organelles or background). Finally, we tested the performance of ilastik on the same image sets used to evaluate the performance of our model (dataset test final, Table 1).

### Statistics

Results are provided as mean ± SEM. To test differences in the performance of the model before and after the application of certain steps we performed a one-way repeated-measures ANOVA and after having verified that at least one step changed the algorithm’s performance significantly, we run multiple pairwise paired *t* test applying the Bonferroni correction for setting the significance of *p*-values. For clarity, we present both the original (*p*) and the Bonferroni corrected (p*) *p*-values. To test differences in the performance between two models or between model and humans, we performed paired *t* tests. To measure the correlations strength between two variables, we calculated the Pearson correlation coefficient. *P*-values below 0.05 were regarded as statistically significant and they are provided approximated at the fourth decimal. In graphs, one asterisk indicates statistically significant differences or correlations.

### Generation of a GUI

For making our algorithm easily accessible to everyone we generated a GUI with the widget toolkit Tk using the python interface tkinter (https://docs.python.org/3/library/tkinter.html). The GUI has the purpose of guiding the experimenter through the required steps to conduct the automatic vesicle analysis and offers a tool to display the results. The results are provided in an excel file and include the number of vesicles per image and, for each predicted vesicle, the *x*, *y* coordinates, the nnd and the estimated area. Furthermore, the GUI includes a manual proof-reading tool which allows users to easily add (false negatives) or remove (false positives) predictions from each analyzed image. These manual changes are automatically incorporated in the result excel file.

All documentations and the instructions about how to use the GUI can be found in the README file in the GitHub repository at the address specified below, in Code accessibility.

### Code accessibility

The codes described in the paper for training the classifiers and for using the GUI are freely available online at: https://github.com/Imbrosci/synaptic-vesicles-detection. Beyond the source codes, a README file, a requirements.txt file as well as the weights of the trained models are also available at the same address. The codes used for data analysis are freely available online at: https://github.com/Imbrosci/synaptic-vesicles-detection-extra. All codes are available as Extended Data 1.

10.1523/ENEURO.0400-20.2021.ed1 Extended Data 1 Codes and README files. Code files used for training the classifiers and for using the GUI: • performance_checker_(ilastik).py (it has the same function as performance_checker.py but on results from ilastik). • density_to_probability_transformer.py (it transforms density images from the Cell Counting workflow of ilastik into probability maps and save them); • performance_checker.py (it evaluates the performance of the algorithm by calculating true positives, false positives and false negatives using human annotations as ground truth); • ROC_AUC_calculator.py (it generates the ROC curve and calculates the AUC score from the first vesicle classifier); Codes used for the analyses: • running_analysis.py (the execution of this code launches the GUI). • Gui_vesicle_detection.py (it is needed to generate the GUI and to conduct image analysis, result visualization and proof-reading); • first_classifier_training.py and second_classifier_training.py (they contain the codes used to train the first and the refinement classifier and to evaluate their performance on the training and validation datasets); • CNNs_GaussianNoiseAdder.py (it contains the two CNNs and some lines to add Gaussian noise to the training dataset as data augmentation strategy); Download Extended Data 1, ZIP file.

## Results

### Evaluation of the algorithm

To develop an algorithm for the automated recognition of synaptic vesicles, we created, as a first step, a vesicle classifier based on CNNs. The model consists of four convolutional layers followed by one 2 × 2 max pooling layer and three fully connected layers (Fig. 1*A*). To train the CNN we generated a large dataset of labeled image patches obtained from micrographs of hMFBs or from small hippocampal synapses either containing or not containing a synaptic vesicle. The training dataset was then further split (75%/25%) to perform four rounds of cross-validation.

First, we evaluated the effect of tuning some hyperparameters on the performance of the model. Specifically, we tried to vary the size of the convolutional filters (5 × 5, 7 × 7, and 9 × 9 pixels) as well as the number of the filters for the first, second, third and fourth convolutional layer: 8–16–32–64, 16–32–64–128, or 32–64–128–256. We selected the model hyperparameters with the highest average four-fold cross-validation performance (lowest average loss and highest F1-score). This model turned out to have convolutional filters of 7 × 7-pixel size and 16–32–64–128 filters (for the first, second, third, and fourth layer, respectively; Fig. 1*A*). Subsequently, we used the entire training dataset (dataset train 1, Table 1) to train a model with the chosen hyperparameters and evaluated its performance on a test dataset generated with the same procedure as the training dataset (from labeled image patches) but from different micrographs (dataset test 1, Table 1). Figure 1*B–F* showed the loss, accuracy, precision, recall, and F1-score for both training and test datasets. The loss rapidly decreased, while the other measurements rapidly increased within the first few epochs in both training and test dataset. Between epochs 10 and 25 the performance of the model clearly reached a plateau (Fig. 1*B–F*). We further calculated the receiver operating characteristic (ROC) curve (Fig. 1*G*) on the test dataset. The area under the ROC curve (AUC) was 96.1%. All together these statistics indicate that our model has a very strong predictive power in this image classification task. We selected the weights from the epoch achieving the highest F1-score on the test dataset (dataset test 1, Table 1) and after which we observed an increase in the loss in three consecutive epochs (epoch 21).

To have a visual confirmation of the classification results, we selected 39 image patches either containing or not containing a vesicle from our test dataset (Fig. 1*H*). The number in the first square bracket, on top of each image, represents the label assigned manually (0 = no vesicle, 1 = vesicle), whereas the number in the second square brackets represents the label predicted by the classifier. Our classifier outputs the probability of a patch to contain a vesicle which was then converted into 0 or 1 with a cutoff value of 0.5. As can be seen by comparing the manual labels and predictions, the great majority of the images were classified correctly by our model.

**Figure 2.**
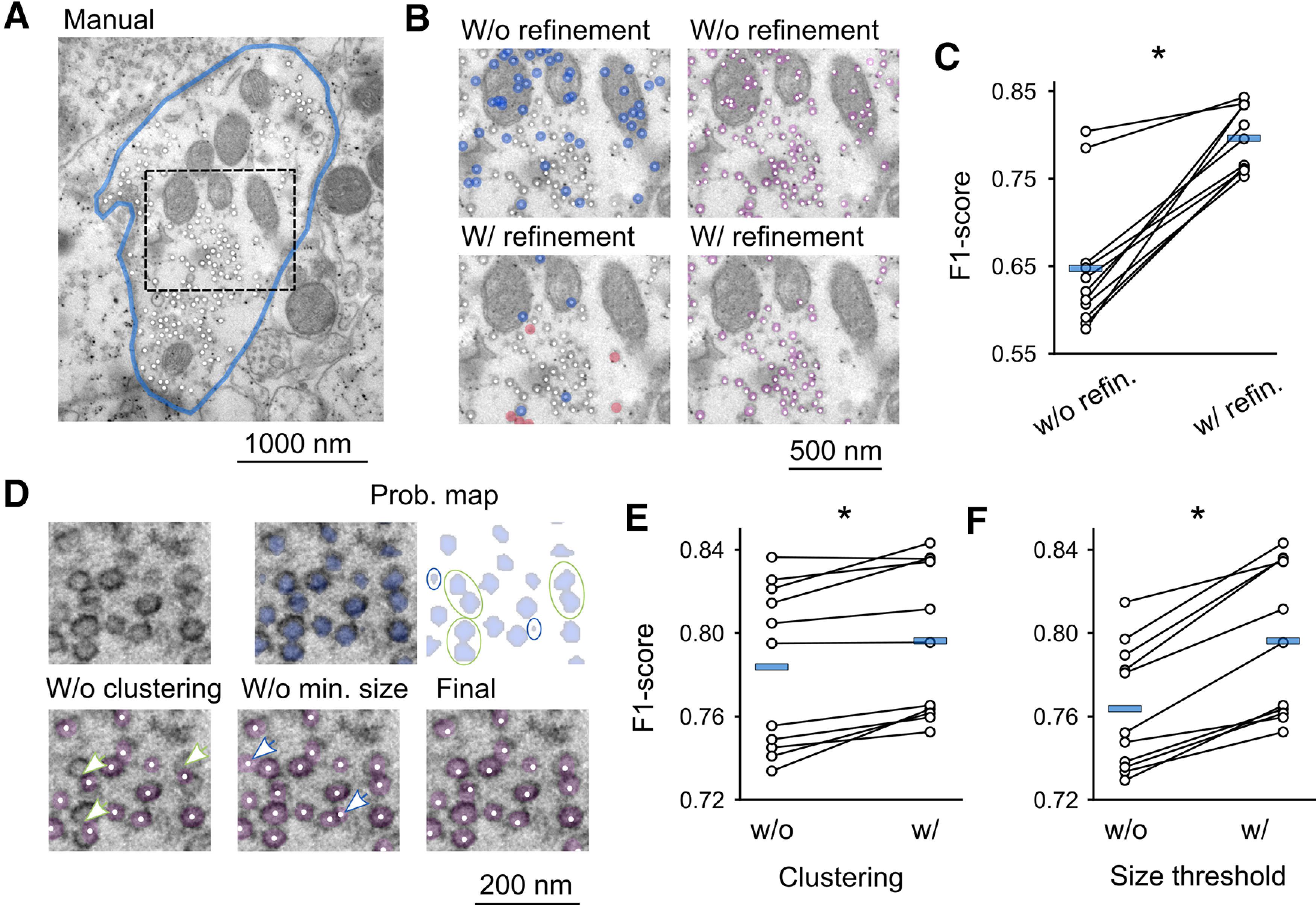
Evaluation of single steps built in the algorithm. ***A***, Micrograph showing a hMFB, from a chemically-fixed acute hippocampal slice, with all manually detected vesicles tagged by the white dots. Only vesicles belonging to the synaptic terminal delimited by the blue line were manually labeled and predicted. ***B***, Magnification of the rectangular area shown in ***A*** with vesicles predicted by the algorithm. The vesicles were predicted either without (top), or with the contribution of the refinement CNN (bottom). On the left, the positions of the predicted vesicles are tagged by the white dots and false positives and false negative are marked by the semi-transparent blue and red circles, respectively. On the right, beyond the position of the predicted vesicles (white dots), the estimated vesicles areas are also represented as overlaid semi-transparent pink mask. ***C***, F1-score without and with the contribution of the refinement CNN. ***D***, EM image of a small portion of a hMFB (top, left), from a chemically-fixed acute hippocampal slice, same portion overlaid with the probability map generated by the first CNN as semi-transparent blue mask (top, middle), probability map alone (top, right), the green open circles point at three erroneously merged vesicles before clustering, while the two blue circles point at two clusters falling below the threshold size for being considered as vesicles. Vesicles detected without clustering-based segmentation (bottom, left), the arrows point at three merge errors. Vesicles detected without setting the size threshold for excluding very small clusters (bottom, middle), the arrows point at two false positives. Vesicles detected after implementing both clustering-based segmentation algorithm as well as after the threshold for excluding too small clusters (bottom, right), note that here both errors types are eliminated. ***E***, F1-score without and with the contribution of the clustering-based segmentation algorithm and of (***F***) the size threshold for excluding very small clusters. * in ***C***, ***E***, and ***F*** indicate a *p* value < 0.05.

Next, we tested the performance of our algorithm on 11 electron micrographs of a hMFB containing hundreds of vesicles (subset of dataset test final, Table 1) and compared the results to human labeled data (Fig. 2*A*). To this end, we incorporated the vesicle classifier in a sliding window algorithm and run thereafter a connected-component labeling and a k-means clustering algorithm as described in detail in Materials and Methods. Figure 2*B*, top, showed that this approach was sufficient to detect the great majority of the vesicles, corroborating the high sensitivity of the model. However, a not negligible number of false positives were present, especially within intracellular organelles, such as mitochondria, or along synaptic membranes. To reduce the number of false positives we let the detected vesicles to be evaluated a second time by the refinement classifier (for details, see Materials and Methods). The weights selected for performing this second round of prediction derived from the epoch achieving the highest F1-score on the test dataset (dataset test 2; epoch 47th). This “double” prediction eliminated the great majority of false positives, especially within mitochondria and other intracellular organelles (Fig. 2*B*, bottom) causing a large increase in the precision of the model. At the same time, the recall was affected to a lesser extent and remained relatively high. Overall, after the prediction by the second CNN, the F1-score of the model improved significantly (*p* < 0.0001, p* < 0.0001; Fig. 2*C*; Table 2).

**Table 2:** Improvement of the model performance by applying additionally the refinement classifier and postprocessing steps

Data	Precision	Recall	F1-score
Without refinement	49.62 ± 2.87%, *n* = 11	95.78 ± 0.60%, *n* = 11	64.73 ± 2.32%, *n* = 11
Without clustering	78.58 ± 1.17%, *n* = 11	79.02 ± 1.93%, *n* = 11	78.38 ± 1.18%, *n* = 11
Without size threshold	71.63 ± 1.12%, *n* = 11	82.78 ± 1.80%, *n* = 11	76.38 ± 0.90%, *n* = 11
Final	77.87 ± 1.15%, *n* = 11	82.34 ± 1.87%, *n* = 11	79.63 ± 1.11%, *n* = 11

Performance of the model (precision, recall, and F1-score) on images of hMFBs from chemically-fixed acute hippocampal slices (subset of dataset test final) without refinement classifier, without clustering-based segmentation, without removal of too small clusters, and with all steps included. Data are presented as mean ± SEM.

A clustering-based segmentation algorithm is likely to be effective in reducing merge errors (which produce false negatives). However, it may also cause an increase in false positives because of split errors. Therefore, we wanted to confirm if our clustering strategy, applied after the first CNN, improved the performance of the algorithm. Our results showed that adding the k-means clustering algorithm to our model increased significantly the F1-score (*p* = 0.0016, p* = 0.0049; Table 5). This was because the recall of the model increased to a greater extent with respect to the decrease in the precision (Fig. 2*E*; Table 2). Therefore, we can conclude that the reduced merge errors exceeded the few split errors generated by this strategy. An example showing merge errors eliminated by our clustering-based segmentation algorithm is displayed in Figure 2*D*, before clustering: bottom, left panel; after clustering: bottom, right panel, see arrows. Finally, we evaluated the effect of setting a threshold to exclude too small clusters from being considered as vesicles. Our analysis showed that introducing this threshold improved significantly the F1-score of the model (*p* < 0.0001, p* < 0.0001; Table 5). This was because of a marked increase in the precision of the model without a significant effect on the recall (Fig. 2*F*; Table 2). An example showing false positives eliminated by the application of a threshold setting a minimal cluster dimension is displayed in Figure 2*D*, without threshold: bottom, middle panel; with threshold: bottom, right panel, see arrows.

**Figure 3.**
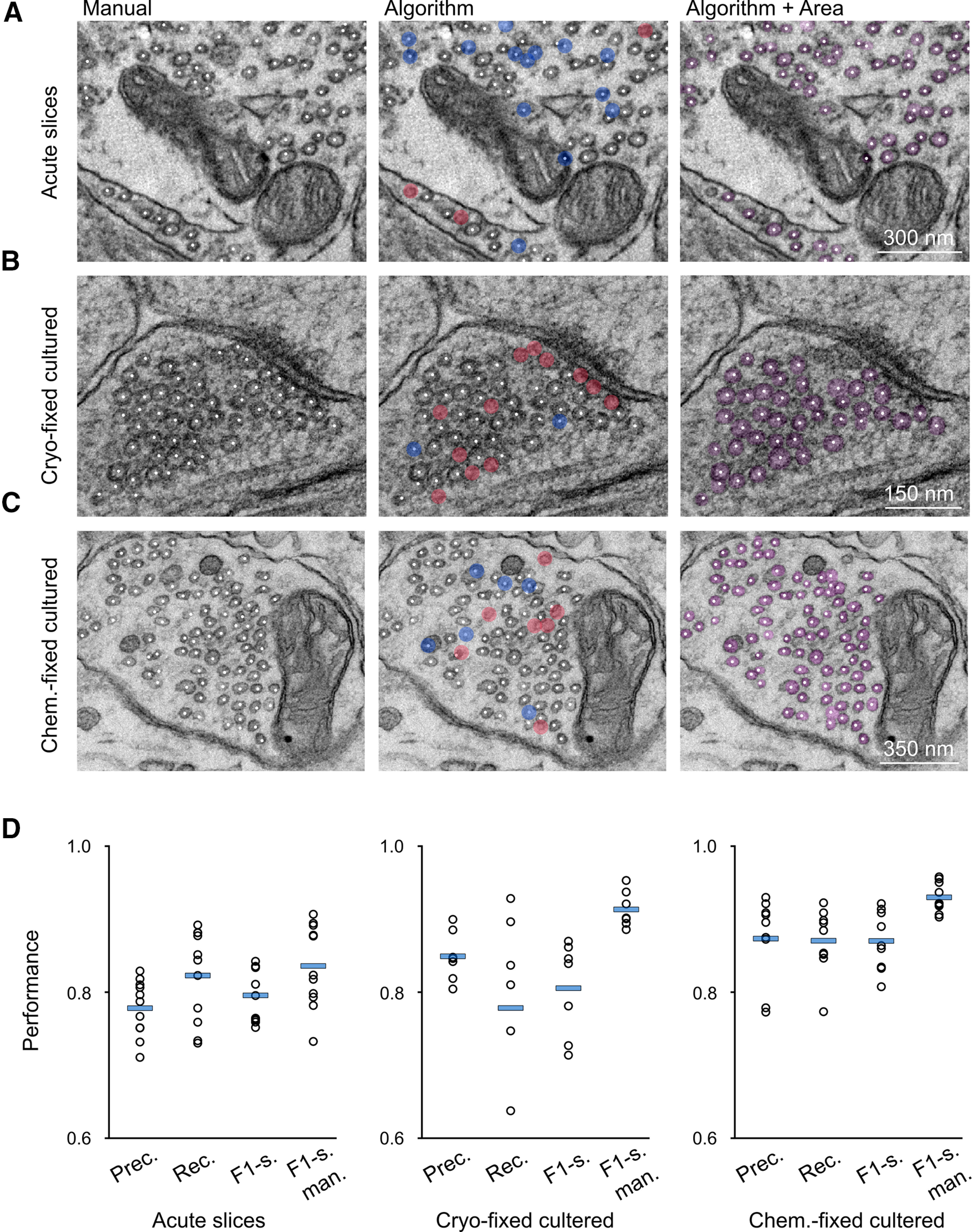
Evaluation of the performance of the algorithm on different sample preparations**. *A***, Portion of a micrograph of a hMFB from a chemically-fixed acute hippocampal slice with all manually detected vesicles tagged by the white dots (left), with all predicted vesicles tagged by the white dots and false positives and false negatives marked by the semi-transparent blue and red circles, respectively (middle), and with all predicted vesicles tagged by the white dots and their estimated areas represented by the overlaid semi-transparent pink mask (right). Same as in ***A***, but here, the micrographs show small hippocampal synapses from (***B***) a cryo-fixed and a (***C***) chemically-fixed neuronal culture. ***D***, Precision, recall, F1-score of the algorithm for the three different sample preparations and F1-score obtained by comparing the results from the two human-based analysis (F1-s. man). For this analysis, only vesicles belonging to one synaptic terminal were manually labeled and predicted. Extended Data Figure 3-1 shows the comparison between synaptic vesicle count detected by humans and by the algorithm. Extended Data Figure 3-2 shows an example of synaptic vesicles detection using ilastik.

Next, we tested the performance of our algorithm on images from different preparations and hippocampal synapses (dataset test final, Table 1; Fig. 3*A–C*).

10.1523/ENEURO.0400-20.2021.f3-2 Extended Data Figure 3-2 Example of synaptic vesicles detection using ilastik. ***A***, Raw micrograph (left), probability map (middle), and segmentation map (right) of a hMFB from a chemically-fixed acute hippocampal slice. The hMFB was isolated by applying a black mask on the surrounding. The probability map was obtained by converting the density image produced by the Cell Density Counting workflow. The segmentation map was then obtained using the Object Classification (inputs: raw data, pixel prediction map) workflow. ***B***, On the left, a portion of the micrograph in ***A***, with all manually detected vesicles tagged by the white dots. On the right the same image, with all the vesicles predicted by ilastik tagged by the dots. The correctly guessed vesicles (true positives) are represented in white, the wrongly predicted vesicles (false positives) in blue and the missed vesicles (false negatives) in red. Download Figure 3-2, TIF file.

The number of vesicles detected by the algorithm was similar to the number of manually detected vesicles in all three preparations (Extended Data Fig. 3-1). The performance of the model was also relatively high (Table 3), although there were differences between preparations: the precision was higher in neuronal cultures with respect to acute slices, while the recall was highest in chemically-fixed and lowest in cryo-fixed neuronal cultures (Fig. 3*D*). To better interpret the quality of these results we also evaluated the difference between human-based analysis conducted by two researchers, independently. When we compared the results from the two human-based analysis with each other (considering the manual analysis of either one or the other researcher as ground truth) we obtained F1-scores which were statistically significantly higher than the results obtained by the algorithm (*p*-values for differences in F1-score, acute slices: *p* = 0.0004, cryo-fixed: *p* = 0.0068, chemically-fixed: *p* = 0.0004; Table 5). Nonetheless, the F1-scores of human analysis were only a few % points higher with respect to the result of the algorithm (from +3.96% in acute slices to a maximum of +10.52% in cryo-fixed neuronal cultures) and they were lower than 100% (see Table 3), suggesting that a margin of uncertainty may be inevitable since present in analyses conducted by humans.

**Table 3:** Evaluation of the model performance on images from different preparations

Data	Precision	Recall	F1-score (alg.)	F1-score (human)
Acute slices	77.87 ± 1.15%, *n* = 11	82.34 ± 1.87%, *n* = 11	79.63 ± 1.11%, *n* = 11	83.59 ± 1.71%, *n* = 11
Cryo-fixed n.c.	85.10 ± 1.30%, *n* = 7	78.13 ± 4.69%, *n* = 7	80.82 ± 2.38%, *n* = 7	91.33 ± 0.93%, *n* = 7
Chemically-fixed n.c.	87.53 ± 1.96%, *n* = 9	87.24 ± 1.53%, *n* = 9	87.22 ± 1.37%, *n* = 9	92.99 ± 0.70%, *n* = 9

Performance of the model (precision, recall, and F1-score) on images of hMFBs from chemically-fixed acute hippocampal slices (acute slices) and of small hippocampal synapses from either cryo-fixed or chemically-fixed neuronal cultures (cryo-fixed n.c. and chemically-fixed n.c., respectively; dataset test final) and F1-score obtained comparing the annotations of two humans with each other. Data are presented as mean ± SEM.

Furthermore, to fill the gap that remains between human and machine performance, we added a proof-reading tool to our GUI which allows to evaluate and correct the predictions done by the algorithm, whenever necessary. Further details to use this function can be found in the README file in the GitHub repository at the address specified in Materials and Methods, Code accessibility.

To further evaluate our results, we compared our algorithm with ilastik, a well-established, machine learning-based tool for (bio)image analysis (Berg et al., 2019). Our strategy to detect synaptic vesicles with ilastik was to use two workflows, sequentially: Cell Density Counting and Object Classification (for details, see Materials and Methods). An example of the results from ilastik can be found in the Extended Data Figure 3-2. The performance of ilastik in detecting synaptic vesicles with the chosen process was statistically significantly lower in comparison to our model for all three preparations (dataset test final, Table 1; *p*-values for differences in F1-score, acute slices: *p* < 0.0001, cryo-fixed: *p* = 0.0003, chemically-fixed: *p* = 0.0056; Tables 4, 5).

**Table 4:** Evaluation of the performance of ilastik and comparison with our model

Data	Pre*cis*ion (ilastik)	Recall (ilastik)	F1-score (ilastik)	F1-score (alg.)
Acute slices	50.60 ± 3.19%, *n* = 11	66.92 ± 4.60%, *n* = 11	56.89 ± 3.36%, *n* = 11	79.63 ± 1.11%, *n* = 11
Cryo-fixed n.c.	63.30 ± 4.52%, *n* = 7	62.62 ± 2.45%, *n* = 7	62.16 ± 2.25%, *n* = 7	80.82 ± 2.38%, *n* = 7
Chemically-fixed n.c.	74.15 ± 3.24%, *n* = 9	71.24 ± 5.97%, *n* = 9	70.48 ± 4.14%, *n* = 9	87.22 ± 1.37%, *n* = 9

Performance of ilastik (precision, recall, and F1-score) on the same images used in Table 3 (dataset test final) and F1-score obtained with our model. Data are presented as mean ± SEM.

**Table 5:** Statistical table

Data type	Compared groups	Test	Results		df
F1-score (%); Fig. 2	Final vs no refin., no cluster, no size thr.	Repeated-measures ANOVA	*F* = 40.33		3
Data type	Compared groups	Test	Confidence level		df
			95%	Bonferroni corr.
F1-score (%); Fig. 2	Final vs no refin.	Paired *t* test	10.44% to 19.36%	9.18% to 20.62%	10
F1-score (%); Fig. 2	Final vs no cluster	Paired *t* test	0.60% to 1.90%	0.41% to 2.08%	10
F1-score (%); Fig. 2	Final vs no size thr.	Paired *t* test	2.34% to 4.15%	2.08% to 4.41%	10
F1-score (%), acute slices; Fig. 3	Algorithm vs humans	Paired *t* test	−5.67% to −2.25%	-	10
F1-score (%), Cry. fix. n.c.; Fig. 3	Algorithm vs humans	Paired *t* test	−16.89% to −4.15%	-	6
F1-score (%), Che. fix. n.c.; Fig. 3	Algorithm vs humans	Paired *t* test	−8.10% to −3.45%	-	8
F1-score (%), acute slices	Algorithm vs ilastik	Paired *t* test	16.20% to 29.27%	-	10
F1-score (%), Cry. fix. n.c.	Algorithm vs ilastik	Paired *t* test	12.42% to 24.89%	-	6
F1-score (%), Che. fix. n.c.	Algorithm vs ilastik	Paired *t* test	6.46% to 27.01%	-	8
Vesicle count; Fig. 6	Algorithm vs humans	Pearson corr.	0.7288 to 0.9494	-	42
Vesicle nnd; Fig. 6	Algorithm vs humans	Pearson corr.	0.2838 to 0.8309	-	42
Vesicle area; Fig. 6	Algorithm vs humans	Pearson corr.	0.4979 to 0.8949	-	42

### Evaluation of the robustness of the algorithm to noise and changes in contrast

Next, we evaluated the effect of adding Gaussian noise or varying the image contrast on the performance of our model. We first added artificial Gaussian noise to six different electron micrographs (three of hMFBs from chemically-fixed acute hippocampal slices and three of small hippocampal synapses from chemically-fixed neuronal cultures; subset of dataset test final, Table 1). The noise was applied on images with the range of pixel values normalized to 0–1. We used a Gaussian distribution with zero mean and gradually increase SD (σ).

**Figure 4.**
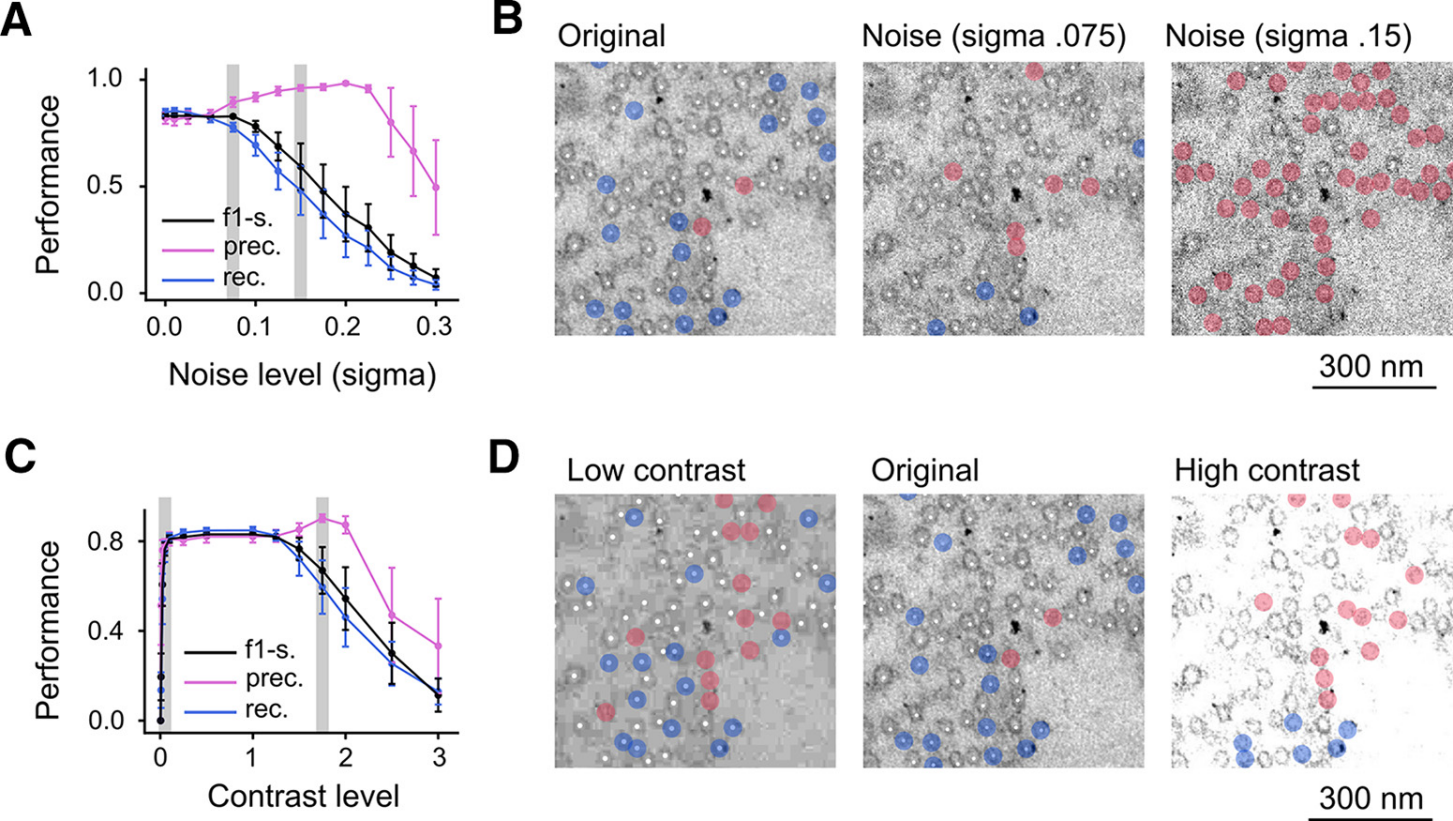
Performance of the model with different levels of noise and contrast. ***A***, Precision (pink), recall (blue), and F1-score (black) at increasing noise levels. ***B***, Portions of micrograph of a hMFB with increasing level of noise (from left to right) with all predicted vesicles tagged by the white dots. The level of noise in the images in the middle and on the right is marked by the gray rectangles in ***A***. ***C***, ***D***, Same as in ***A***, ***B***, but instead of noise, different levels of contrast were tested. The contrast level in the images on the left (low contrast) and on the right (high contrast) is marked by the gray rectangles in ***C***. For this analysis, only vesicles belonging to one synaptic terminal were manually labeled and predicted. On ***A***, ***C***, the dots represented the mean and the bars the SEM.

Up to a relative high level of noise (σ 0.2), the model’s precision improved, while increasing the noise caused a decline in the model’s recall. This was because of a large increase in the number of false negatives. This suggests that, on noisy images, the model is very conservative in deciding about the presence of a vesicle. Above a certain level of noise both precision and recall dropped. Because of this different behavior between precision and recall, the F1-score declined relatively slowly (Fig. 4*A*,*B*).

We then artificially decreased or increased the level of contrast in the same electron micrographs used in Figure 4*A*,*B*. We observed a large plateau in model performance so that it was only marginally affected in images with a contrast level between 0.05 and 1.5 times the level of the original images. In general, the F1-score remained almost unchanged for a large range of contrasts demonstrating the robustness of our model to changes in this parameter (Fig. 4*C*,*D*).

### Evaluation of the robustness of the algorithm on publicly available images

Finally, to test the limits of our model, we evaluated its performance on images with pixel size ranging from 0.69 to 5.15 nm, coming from different sample preparations, different species, and acquired with different microscopes in other laboratories (dataset external, Table 1).

**Figure 5.**
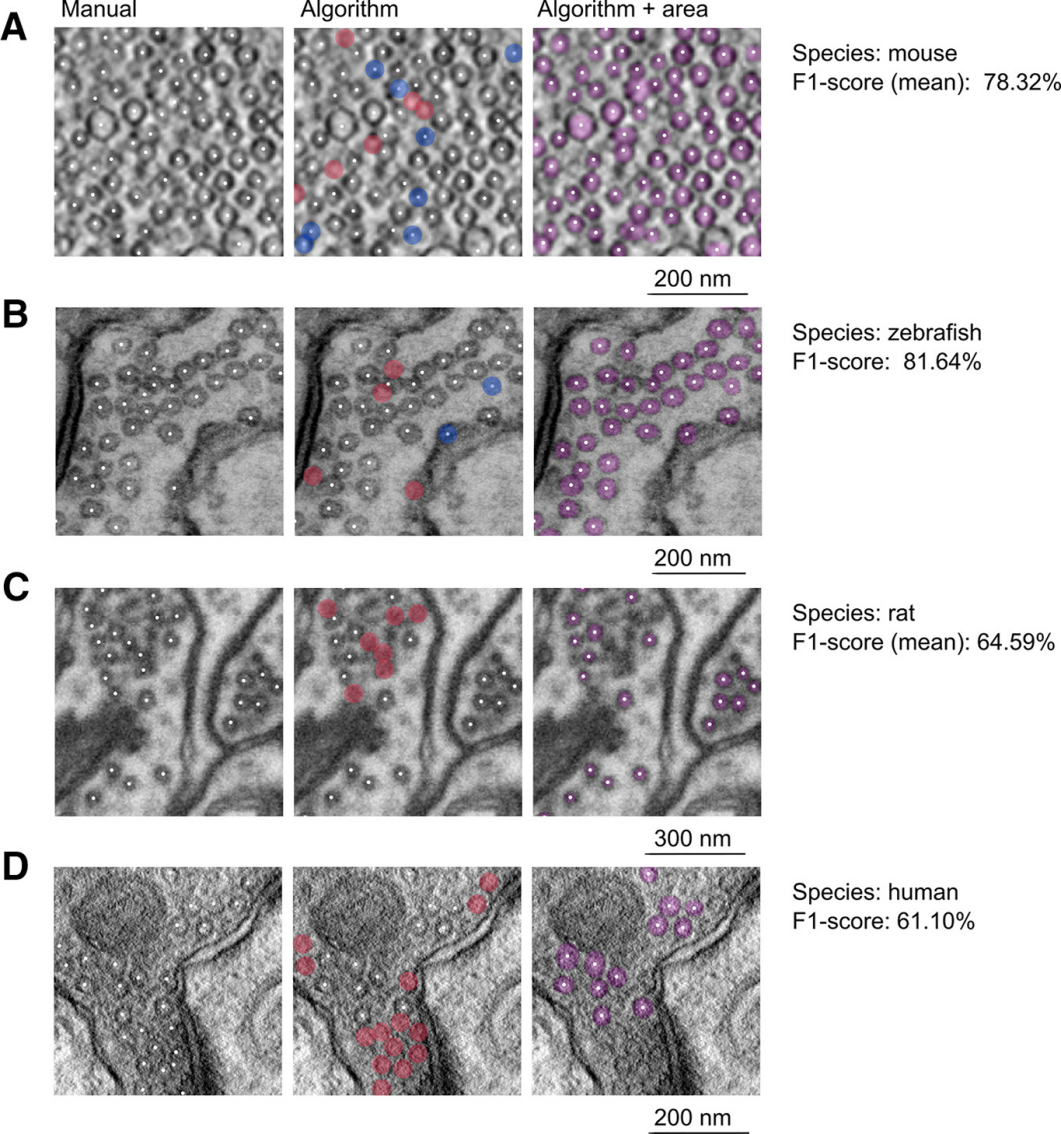
Performance of the algorithm on images available online. ***A–D***, Portion of micrographs containing synaptic vesicles with all manually detected vesicles tagged by the white dots (left), with all predicted vesicles tagged by the white dots and with false positives and false negatives marked by the semi-transparent blue and red circles, respectively (middle), and with all predicted vesicles tagged by the white dots and their estimated areas represented by the overlaid semi-transparent pink mask (right). The image in ***A***, is a portion of a virtual section of an electron tomogram from a cryo-fixed mouse hMFB (Imig et al., 2020); the image in ***B*** belongs to a synapse from a chemically-fixed zebrafish optic tectum (http://cellimagelibrary.org/images/6230). ***C***, A portion of a synapse from a serial block face scanning EM (https://github.com/NeuroMorph-EPFL/NeuroMorph/tree/master/NeuroMorph_Datasets/EM_stack). ***D***, A virtual slice of an electron tomogram of a chemically-fixed human synapse from the temporal lobe neocortex. Images in ***A***, ***B***, ***D*** were obtained with a transmission EM, whereas the image in ***C*** was obtained with a scanning EM. For this analysis, all vesicles present in the images were manually labeled and predicted.

We tested two images from virtual sections of an electron tomogram of cryo-fixed mice synapses (pixel size = 5.15 nm; Imig et al., 2020; see their videos S1 and S2) and obtained a precision of 73.93 ± 7.45%, a recall of 83.68 ± 0.73% and an F1-score of 78.32 ± 4.54% (Fig. 5*A*), the vesicle count detected manually and by the model was 534.75 ± 283.25 and 580.5 ± 267.50, respectively; one transmission EM image from a chemically-fixed zebrafish synapse (pixel size = 1.61 nm, http://cellimagelibrary.org/images/6230) and obtained a precision of 86.21%, a recall of 77.54% and an F1-score of 81.64% (Fig. 5*B*), the vesicle count detected manually and by the model was 129 and 116, respectively; six images from serial block face scanning EM of chemically-fixed synapses [pixel size = 5 nm (Jorstad et al., 2015); https://github.com/NeuroMorph-EPFL/NeuroMorph/tree/master/NeuroMorph_Datasets/EM_stack] and obtained a precision of 81.52 ± 2.10%, a recall of 53.78 ± 1.39% and an F1-score of 64.59 ± 1.44% (Fig. 5*C*), the vesicle count detected manually and by the model was 133.25 ± 14.17 and 86.67 ± 7.94, respectively, and finally a virtual section from an electron tomogram of chemically-fixed human synapses (pixel size = 0.69 nm; Rollenhagen et al., 2020; see their Movie 2) obtaining a precision of 86.76%, a recall of 49.08% and an F1-score of 61.10% (Fig. 5*D*), the vesicle count detected manually and by the model was 67 and 34, respectively. The precision of the model was similar across all images and comparable to the one obtained with our own images. However, one limitation of our algorithm was that the recall was more variable, mainly because of the fact that vesicles that were not sharp or that did not have a clearly visible membrane were often false negatives.

### Parameters provided by the algorithm

**Figure 6.**
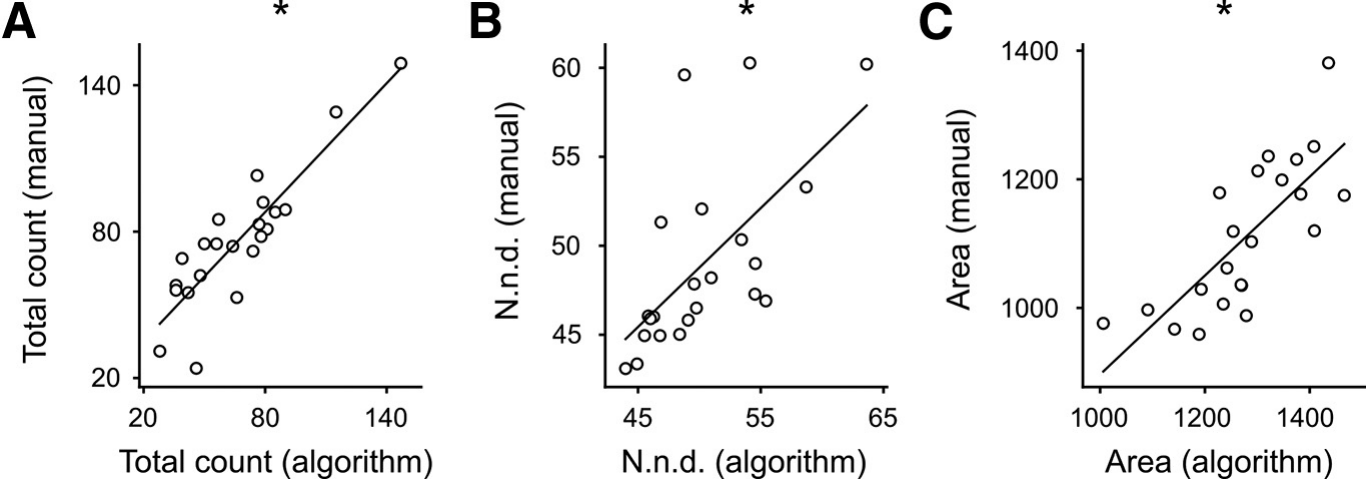
Correlations of parameters obtained by human analysis or by the algorithm. Correlations between algorithm and human results for (***A***) total vesicle count, (***B***) nnd, and (***C***) estimated vesicle area. Each dot represents the average value for an image. The black lines represent the linear regressions. * indicate a *p* value < 0.05.

Beyond the vesicle count and the position of each detected vesicle, our algorithm provides the nnd and the estimated area of each vesicle. We compared the values obtained by our system with the ones measured manually in 22 images of small hippocampal synapses (10 from cryo-fixed and 12 from chemically-fixed neuronal cultures; subset of dataset test final, Table 1) and observed significant correlations for all three parameters (Fig. 6*A–C*, vesicle count: *r* = 0.88 *p* < 0.0001, nnd: *r* = 0.63, *p* = 0.0015, area: *r* = 0.76, *p* < 0.0001; Table 5).

## Discussion

In the present study we present a successful application of deep CNNs for the automated recognition of nanoscale organelles (synaptic vesicles) in EM images.

Recent studies showed that the distribution of synaptic vesicles can underlie neuromodulation (Patzke et al., 2019, 2021) and synaptic plasticity (Rey et al., 2020; Orlando et al., 2021; Reshetniak and Rizzoli, 2021). Therefore, automating synaptic vesicles detection constitutes an important tool for researchers interested in synaptic function and plasticity.

Deep neural networks are acquiring growing importance in many aspects of our lives. They contribute to the extraordinary advances of many digital applications, such as automatic speech recognition, natural language processing, object recognition, and cancer diagnosis just to mention a few (Shrestha and Mahmood, 2019). CNNs are a class of deep neural networks heavily employed in the field of computer vision. Thanks to their unique architectures, inspired by the visual cortex, they achieved unpreceded results in visual tasks ranging from image classification and object detection to autonomous driving (Rawat and Wang, 2017).

CNNs have already found different applications in the field of neuroanatomy. In particular, they showed to achieve very high accuracy in the segmentation of neuronal structures and they have been employed for the computation of 3D reconstruction of neuronal micro-circuitry in connectomics studies (Cireşan et al., 2012; Ronneberger et al., 2015; Januszewski et al., 2018). Despite these advanced applications of CNNs, the automated segmentation of synaptic vesicles remains a challenge because of vesicle size, which is often smaller than the z resolution of 3D reconstructions. A recent application of the CDeep3M software (Steinkellner et al., 2021) seems to be nowadays the only tool capable to localize synaptic vesicles but it still requires a re-training of the segmentation algorithm.

We therefore devoted our effort in the development of a ready-to-use software specialized in the detection of synaptic vesicles from transmission EM images.

For training our CNN-based vesicle classifier we used ∼90 × 90 nm images patches, the majority of which was obtained from micrographs of hMFBs and a smaller portion from images of hippocampal cultured neurons. hMFBs are a particular type of synapse showing a peculiar form of presynaptic plasticity (Nicoll and Schmitz, 2005). Each hMFB, similarly to other large synapses (e.g., neuromuscular junctions, calyx of held, cerebellar mossy fibers), contains up to thousands synaptic vesicles thus constituting an excellent model for establishing the automation of synaptic vesicles detection.

When we evaluated the performance of our vesicle classifier on the test dataset, consisting of patches from a different set of images, either containing or not containing a synaptic vesicle, we obtained a predictive power above 96% (Fig. 1). This result suggests that our model efficiently learned to extract relevant features for predicting the presence or the absence of a vesicle in an image.

The network architecture of our vesicle classifier was inspired by LeNet-5 (Le Cun et al., 1989), a pioneer image classification CNN that became famous for its ability to automatically recognize handwritten digits, and slightly modified to increase performance (Fig. 1). Despite the fact that, recently, more sophisticated CNN architectures have been developed and employed in neuron segmentation studies (Cireşan et al., 2012; Ronneberger et al., 2015; Januszewski et al., 2018), here we show that the relatively simple architecture we chose is sufficient for detecting structures which are largely homogeneous in size and shape as synaptic vesicles.

To detect and localize multiple synaptic vesicles from an entire image from a hMFB, we incorporated the vesicle classifier in a sliding window detection algorithm. Despite sliding window detectors are generally highly computationally expensive, the computational cost of our algorithm was acceptable because small clear synaptic vesicles have a very similar size and shape and therefore, we need to use just a single window size to slide through the image.

When tested on micrographs of mossy fibers presynaptic terminals, our CNN model, combined with a connected-component labeling and a k-means clustering algorithm, effectively detected and localized the great majority of synaptic vesicles. However, we could still systematically observe false positives especially within mitochondria and along membranes. This was expected since these organelles contain vesicular structures which strongly resemble synaptic vesicles. We managed to largely overcome this limitation by implementing a second network, so called refinement classifier, which functions as a check-point to confirm or reject all vesicles predicted as such by the first CNN. Thanks to this additional step, we managed to eliminate a large portion of false positives and significantly improved the performance (F1-score) of our model (Fig. 2*B*,*C*; Table 2).

We also tested our clustering-based segmentation algorithm and the threshold setting for excluding too small clusters and confirmed the importance of these steps, which follow the first CNN, in improving the performance of our model. (Fig. 2*D–F*; Table 2).

After the evaluation of all single steps built in our model, we tested its overall performance on chemically-fixed acute slices, cryo-fixed, and chemically-fixed neuronal cultures from the mouse hippocampus. The model performed well in all three sample preparations, reaching a mean F1-score just below 80% (∼79.6%) in hMFBs from acute slices, ∼80.8% in cryo-fixed neuronal cultures and ∼87.2% in chemically-fixed neuronal cultures (Fig. 3; Table 3). In considering the performance achieved by our model, we should point out that even manual analyses are likely to have some margin of error. Indeed, when we compared the human annotations performed by two postdoctoral researchers, we realized that they did not coincide entirely, but they display marginal differences. This highlights that morphologic manual analyses of this kind are susceptible to human subjectivity. This is likely because of the fact that consistent portion of vesicles are not clearly distinguishable in an electron micrograph, mainly because synaptic vesicles are 3D structures, and the image is a 2D projection of a 3D section.

The uncertainty present in the analysis conducted by humans suggests that it may be impossible to reach a performance near 100%, and it implies that the manually originated training dataset may also not be completely unbiased. In this regard, we want to highlight that, even if our model inevitably inherits the bias present in the manual labels, it will still offer the advantage of using the very same detection strategy for every tested image, making it a very useful tool for groups comparisons (for instance, control vs treatment).

Next, to verify whether our algorithm brings about a substantial improvement in the automate detection of synaptic vesicles with respect to already available tools, we analyzed the same images using ilastik, an interactive machine learning-based tool specialized in (bio)image analysis (Berg et al., 2019). The results of our algorithm were significantly better than the ones obtained with ilastik on the same sets of images (Table 4). Therefore, despite ilastik remains a very useful and flexible tool for a large variety of image analyses, our solution is superior on the specific task of detecting synaptic vesicles.

Finally, to offer the possibility to refine the automatic analysis, we also provide a proof-reading tool to easily add-delete false negatives and positives, respectively.

Next, we evaluated the robustness of our model in face of changes in noise and contrast. Interestingly, the precision of the model improved when Gaussian noise was added to the original micrographs (up to a relatively high level of noise, σ =0.2). The introduction of noise caused also an increase in false negatives, as seen by the decline in recall. The sum of these two effects caused the overall model performance (F1-score) to be relatively constant up to a low-moderate level of noise (σ 0.075) and to then decline (Fig. 4*A*,*B*). Since we consider unlikely that recent image acquisition systems produce images with a noise higher than to the one simulated in this study with a σ ≥ 0.075, we are confident that differences in noise level are unlikely to significantly affect our model. The performance of the model was only marginally affected when tested on images with a large range of contrast levels. As for the noise, changing contrast negatively affected the recall more than the precision. However, in general the F1-score remained almost unchanged in images with contrast level far more extreme that what is usually produced by transmission EM (Fig. 4*C*,*D*). The reported robustness of our model to changes in noise and contrast is likely the result of introducing noise and changes in contrast as data augmentation strategy while training the networks.

As ultimate test, to evaluate to what extent our model performs well, we used images taken from either public repositories or from the Extended Data of two publications (Imig et al., 2020; Rollenhagen et al., 2020; Fig. 5). These included images of synapses from different species, taken at different resolutions and prepared with different protocols. Remarkably, the precision of the model was similarly high in all kind of images tested and comparable with the precision obtained on our own images. However, the recall showed important differences, and it was relatively low in some of the tested images, thereby affecting the F1-score. Based on these results, we can deduce that our algorithm is very precise in detecting vesicles across a broad range of different image types but its efficiency in recognizing vesicles might have consistent variations depending on the vesicle appearance. For instance, in images acquired with a scanning EM, as in Figure 5*C*, false negatives were mainly present if vesicles were not sharp or their lumen was not recognizable. For rendering the application of our model possible also to these cases we still offer the possibility to refine the results with our proof-reading tool or to re-train the model with one own images, by providing in our GitHub repository the source code of the vesicle classifiers and the codes to train them (see README file of the GitHub repository at the address specified in Materials and Methods). Taken together, our results show that our algorithm generalizes well and we are confident that most people working on transmission EM images can directly use the weights from our trained models (the weights can also be found in the public GitHub repository). The main reasons why we believe the model is likely to work on the majority of transmission EM images are the following: (1) it was trained on images from both chemically-fixed and cryo-fixed samples; (2) the shape and dimension of synaptic vesicles varies only marginally across species, brain area and preparation techniques; (3) transmission EM produces images with good resolution allowing to distinguish the membrane delimiting the vesicles as well as their lumen; (4) we included a step to rescale all input images before being evaluated by our CNNs. This allows the model to work with images of different resolutions.

Finally, our algorithm does not only count the number of vesicles, but it also outputs the position, the nnd, and the estimated area for each detected vesicle (Fig. 6). The provided values can be used for measuring many parameters such as synaptic vesicle density, vesicle distribution inside the terminal, and distance from the active zone. These measurements are all important for gaining insight into synaptic function and modulation.

Furthermore, thanks to the provided GUI, our solution has the great advantage of being easy to use by life-science researchers with little programming experience.

It is conceivable that future versions of the algorithm will be trained to further recognize and distinguish other intracellular organelles. The recently developed family of object detection algorithms, R-CNNs, are well suited for achieving these goals. By combining a region proposal network (RPN) with a CNN, they can effectively and accurately localize objects of different size and shape within an image (Girshick, 2015).

In summary, in the present study we developed and evaluated an algorithm to automate the analysis of synaptic vesicles in transmission EM images. We believe that the implementation of this automatic method can strongly increase the throughput of research focusing on synapses structure and function.

## Synthesis

Reviewing Editor: Kristen Harris, The University of Texas at Austin Decisions are customarily a result of the Reviewing Editor and the peer reviewers coming together and discussing their recommendations until a consensus is reached. When revisions are invited, a fact-based synthesis statement explaining their decision and outlining what is needed to prepare a revision will be listed below. The following reviewer(s) agreed to reveal their identity: Forrest Collman.

Most of the prior concerns have been resolved. The following remaining comments should be addressed.

Include a requirements.txt or a conda freeze yaml file in your GitHub repo to help people get versions of the code that you used, rather than getting the latest available which could lead to ‘Code Rot’. For example: When one of the reviewers downloaded the code, the install instructions which worked, but he/she was unable to test the software assumedly because they had a recent mac, and it noted a bug.

Section Vesicles detection using Ilastik: The authors do not mention if post-processing steps are applied. However, as they show in Figure 3 the post-processing step increases the performance of their approach. It would be important to compare the results obtained with Ilastik following similar post-processing steps.

Line 357-359: The weights of the best performing model should not be chosen according to the testing dataset. However, from what could be understood the authors do not report the final performance on this dataset but on another testing set. This strategy should be made clearer in the final version of the paper. Using a table with the datasets used for the different models would improve the clarity of the paper.

Section Evaluation of the robustness of the algorithm on publicly available images: The authors specify that the F1-score is relatively high for all tested types of images. However, the authors claim that they can count the number of vesicles present within an image but have a relatively low recall (48.70% for human synapses). This implies that about 50% of the vesicles are missed by the model. This is not a problem a priori for the implementation but the authors should make this limitation clearer.

Minor Comments:

Line 146: The author should include the number of black patches.

Line 254-264: The author should consider using the mathematical form for the equations. Otherwise, it is difficult to follow.

Section Quantification of the performance of the algorithm: The way the authors treat the TP/FP can be misleading. If multiple predicted annotations are found within the specified distance to ground truth annotations, then one of the predicted annotations should be considered a TP while the others should be considered FPs. This could be better represented by including in the quantification the number of merge and splits.

Line 294: How is the density converted into a probability?

Line 297: The example of segmentation maps from Ilastik could not be found, nor could the quantification of the model performance in Figure 3.

Line 409-410: The F1-score values of the Expert annotators should be reported in Figure 3.

Line 420-422: This sentence does not add to the comprehension of the results and should be removed. This could be included in the provided README file.

Line 433: The authors should add the scores that were obtained with their own implementation to facilitate the reading of the table.

Section Evaluation of the robustness of the algorithm on publicly available images: Please explain how the performance that the authors obtained are not biased by the relatively low number of vesicles in rat/human compared to mouse (see example image in Figure 5). In addition, the authors should report the average number of vesicles per image and the number of missed regions.

Typos:

Line 150/220 The author should consider using the term spatial instead of the position for data augmentation since the only data augmentation used is rotation.

Line 242: Change raw to row.

Line 356: Missing a “.” after 96.1%.

Line 368: Change “compare” to “compared"
