# Erratum: Imbrosci et al., “Automated Detection and Localization of Synaptic Vesicles in Electron Microscopy Images”

**DOI:** 10.1523/ENEURO.0123-22.2022

**Published:** 2022-04-08

In the article, “Automated Detection and Localization of Synaptic Vesicles in Electron Microscopy Images,” by Barbara Imbrosci, Dietmar Schmitz, and Marta Orlando, which was published online on January 4, 2022, the incorrect image was published for Extended Data Figure 3-2 because of a production error. The online version has been corrected.

