# Supplementary figures and images for: Automated Detection and Localization of Synaptic Vesicles in Electron Microscopy Images
Source: eNeuro. 2022 Jan 19;9(1):ENEURO.0400-20.2021. doi: 10.1523/ENEURO.0400-20.2021 (PMC8805189; doi:10.1523/ENEURO.0400-20.2021)

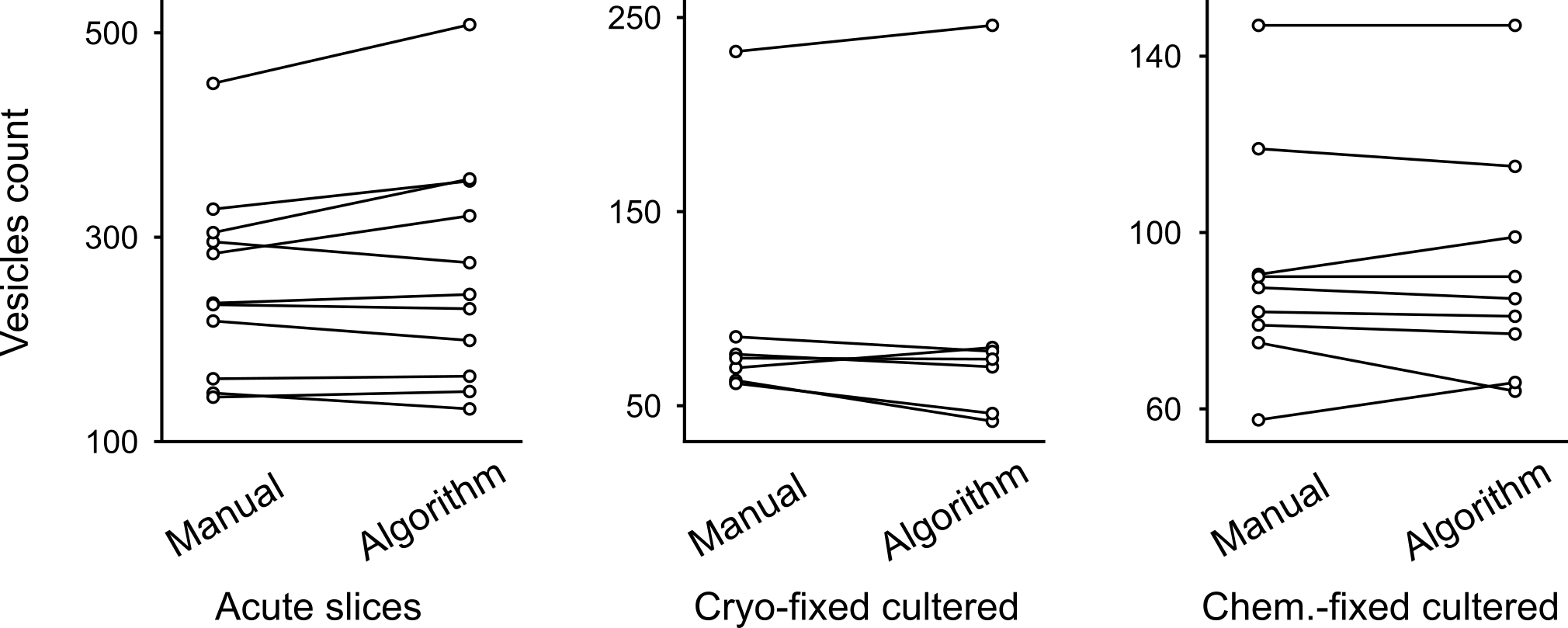

Supplement: Extended Data Figure 3-1 — Comparison between synaptic vesicles count detected by humans and by the algorithm. Number of vesicles detected manually and by the algorithm in micrographs from (A) hMFBs from chemically fixed acute hippocampal slices, (B) small hippocampal synapses from cryo-fixed cultured neurons, and (C) small hippocampal synapses from chemically-fixed cultured neurons. Download Figure 3-1, TIF file. [file enu-eN-MNT-0400-20-s02.tif]

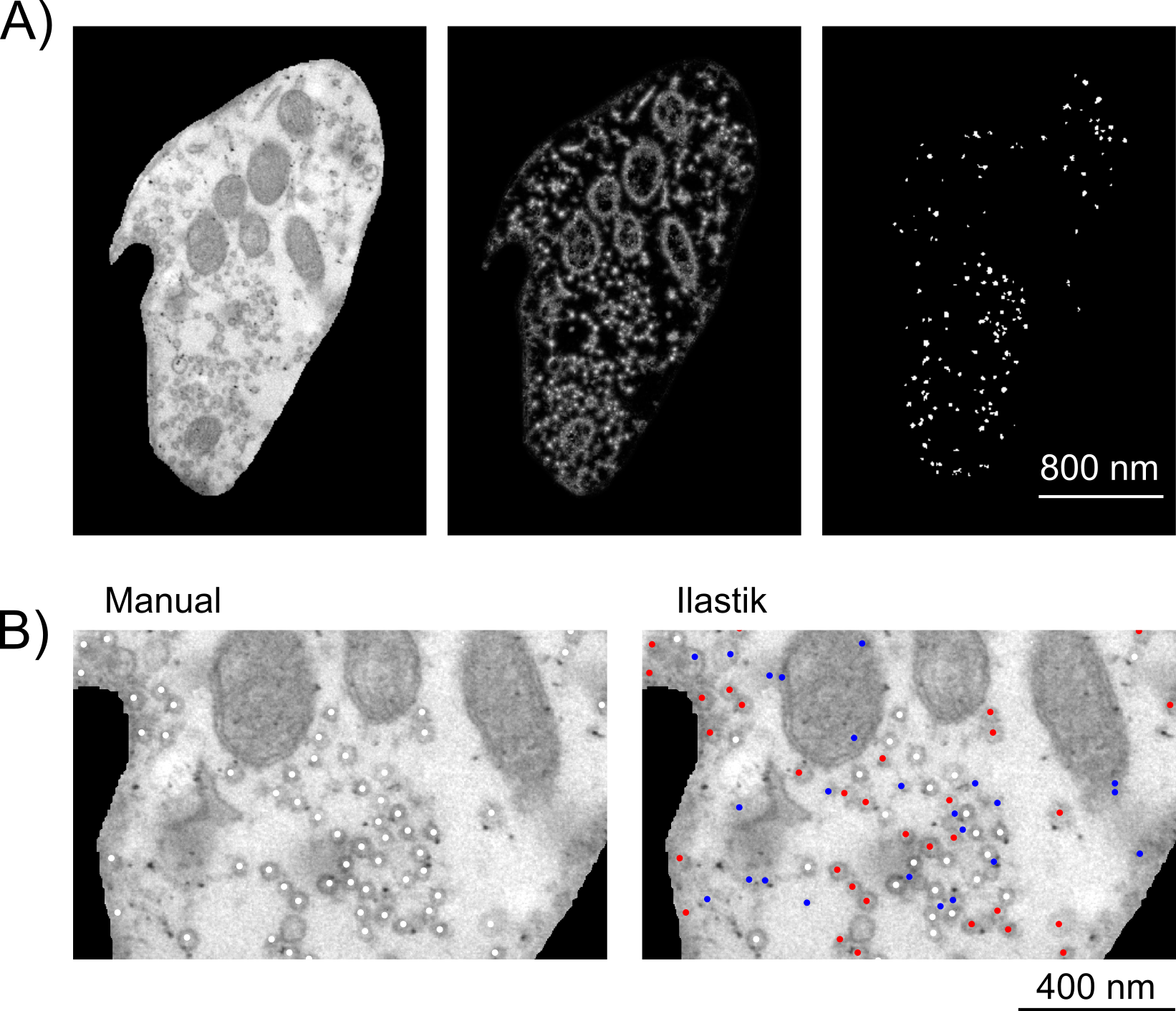

Supplement: Extended Data Figure 3-2 — Example of synaptic vesicles detection using ilastik. A, Raw micrograph (left), probability map (middle), and segmentation map (right) of a hMFB from a chemically-fixed acute hippocampal slice. The hMFB was isolated by applying a black mask on the surrounding. The probability map was obtained by converting the density image produced by the Cell Density Counting workflow. The segmentation map was then obtained using the Object Classification (inputs: raw data, pixel prediction map) workflow. B, On the left, a portion of the micrograph in A, with all manually detected vesicles tagged by the white dots. On the right the same image, with all the vesicles predicted by ilastik tagged by the dots. The correctly guessed vesicles (true positives) are represented in white, the wrongly predicted vesicles (false positives) in blue and the missed vesicles (false negatives) in red. Download Figure 3-2, TIF file. [file enu-eN-MNT-0400-20-s03.tif]
